# Control of growth and development of preantral follicle: insights from *in vitro* culture

**DOI:** 10.21451/1984-3143-AR2018-0019

**Published:** 2018-08-03

**Authors:** José Ricardo de Figueiredo, Laritza Ferreira de Lima, José Roberto Viana Silva, Regiane Rodrigues Santos

**Affiliations:** 1 Laboratory of Manipulation of Oocytes and Preantral Follicles, Faculty of Veterinary, State University of Ceara, Fortaleza CE, Brazil; 2 Biotecnology Nucleus of Sobral (NUBIS), Federal University of Ceara, Sobral, CE, Brazil; 3 Schothorst Feed Research, Lelystad, The Netherlands

**Keywords:** folliculogenesis, *in vitro* development, ovary, preantral follicle.

## Abstract

The regulation of folliculogenesis involves a complex interaction among endocrine, paracrine and autocrine factors. The mechanisms involved in the initiation of the growth of the primordial follicle, i.e., follicular activation and the further growth of primary follicles up to the pre-ovulatory stage, are not well understood at this time. The present review focuses on the regulation and development of early stage (primordial, primary, and secondary) folliculogenesis highlighting the mechanisms of primordial follicle activation, growth of primary and secondary follicles and finally transition from secondary to tertiary follicles. We also discuss the importance of *in vitro* follicle culture for the understanding of folliculogenesis during the preantral phase. Studies suggest that follicular development from primordial to early antral stages is primarily controlled by intra-ovarian ligands but it can also be influenced by many extra-ovarian factors. The control of early folliculogenesis is, therefore, extremely complex because several ligands act through distinct signaling pathways that form sophisticated information networks responding to multiple, often opposing, stimuli. The balance among different stimuli determines follicular survival or death as well as quiescence or activation (growth). The distribution of the ligands and their corresponding receptors varies among follicular compartments and species, and significant changes in gene expression pattern among follicular categories have been reported. Knowing that follicular requirements during early folliculogenesis can be stage-specific and species-specific, *in vitro* culture studies offer an alternative to evaluate single and combined factors during a specific period of follicular development. Herewith we summarize the main findings obtained *in vitro* together with the mechanisms regulating folliculogenesis.

## Introduction

Folliculogenesis is the physiological process of formation, activation, growth and maturation of ovarian follicles. It describes the progression of some small primordial follicles into large preovulatory follicles. The regulation of folliculogenesis involves a complex interaction among endocrine, paracrine and autocrine factors, which in turn affects the steroidogenesis, angiogenesis, basement membrane turnover, oocyte growth and maturation as well as follicular atresia (reviewed by Atwood and Meethala, 2016). It is well known that mammalian ovaries contain from thousands to millions of follicles, whereby about 90% of them are represented by preantral follicles (PFs). The mechanisms involved in the initiation of growth of the primordial follicles, i.e., follicular activation and the further growth of primary follicles up to the pre-ovulatory stage, are not well understood at this time. It is important to emphasize that despite the large number of follicles in the ovary, the vast majority (approximately 99.9%) of them become atretic during their growth and development stages (reviewed by Figueiredo *et al*., 2011).

The *in vitro* follicle culture (IVFC) technology represents a valuable tool to preserve the fertility in individuals subjected to cancer treatment as well as sub- or infertility treatment, to create gamete banks from endangered species and breeds, to complement other reproductive technologies (e.g., *in vitro* embryo production), and to be used as models for studies in reproductive toxicology (reviewed by Figueiredo *et al*., 2011). Furthermore, IVFC provides insights on the control of growth and development of preantral and antral follicles (Cadenas *et al*., 2017). The present review focuses on the regulation and development of early stage (primordial, primary, and secondary) of folliculogenesis highlighting the mechanisms of primordial follicle activation, growth of primary and secondary follicles and, finally, transition from secondary to tertiary follicles. We also discuss the importance of IVFC for the understanding of folliculogenesis during the preantral phase.

## Basic aspects of follicle structure and populations

The ovarian follicle is the functional unit of the ovary composed of an oocyte surrounded by companion somatic cells (granulosa and theca cells). To facilitate the understanding of this review the following follicular classification was adopted: quiescent or dormant follicles represented by the primordial follicles (one layer of flattened granulosa cells around the oocyte) and growing follicles (intermediate: one layer of flattened and cuboidal granulosa cells; primary: one layer of cuboidal granulosa cells, and secondary: two or more layers of cuboidal granulosa cells around the oocyte; Silva *et al*., 2004). However, some authors named dormant follicles the ones containing either one layer of flattened granulosa cells or with flattened and/or cuboidal (Jimenez *et al*., 2016). All preantral follicle categories contain an immature oocyte at the germinal vesicle stage.

## Regulation of folliculogenesis during the preantral follicle phase

**Figure 1:**
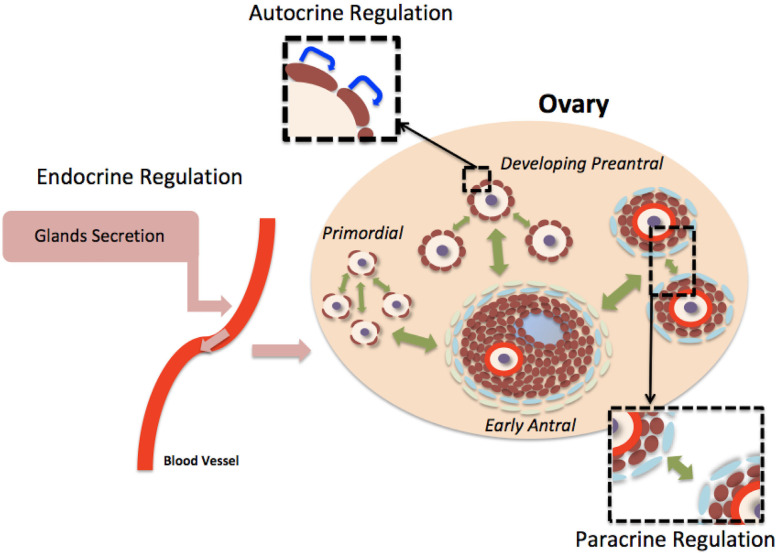
Control of the development of preantral follicle. Follicular development from primordial to early antral stages is primarily controlled by intra-ovarian ligands, but it can also be influenced by many extra-ovarian factors. This process is complex and involves autocrine (blue arrows), paracrine (green arrows) and endocrine (pink arrows) regulations.

**Figure 2:**
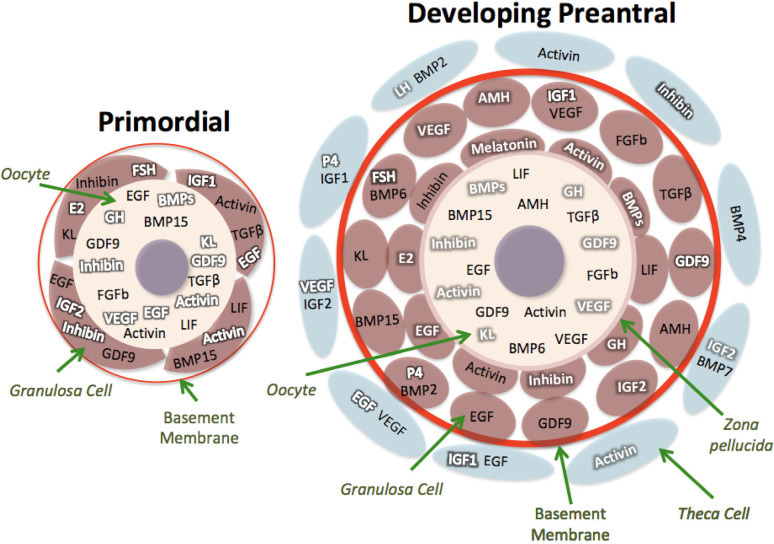
Distribution of some key growth factors and hormones within primordial and developing follicular compartments (oocyte, granulosa and theca cells) in ruminants. Ligands (bold black letter) and receptors (bold white letter). KL - Kit ligand; E2 - 17b-estradiol; FGFb - basic fibroblast growth factor; VEGF - vascular endothelial growth factor; GDF-9 - Growth differentiation factor-9; LIF - leukemia inhibitory factor; GH - growth hormone; EGF - Epidermal growth factor; AMH - Antimullerian hormone; BMP15 - Bone morphogenetic protein 15; BMP6 - Bone morphogenetic protein 6; BMP2 - Bone morphogenetic protein 2; BMP4 - Bone morphogenetic protein 4; IGF1 and IGF2 - Insulin growth factors 1 and 2; TGFβ- tumor growth factor beta; BMPs - Bone morphogenetic proteins superfamily; FSH - Follicle stimulating hormone; LH - Luteinizing hormone.

Folliculogenesis is a highly regulated developmental sequence resulting in the growth and differentiation of the oocyte and associated somatic cells. Capacity of the oocyte to resume meiosis, complete maturation (oocyte maturational competence), undergo successful fertilization, support normal embryo and fetal development and produce healthy offspring (oocyte developmental competence) is gradually acquired as the oocyte develops as the follicles pass through the primordial to the preovulatory stages (reviewed by Figueiredo *et al*., 2011). The production of a good quality oocyte (developmental competence) depends on a fine crosstalk between the oocyte and its surrounding follicular cells that begins during the preantral follicle phase of folliculogenesis. The development of PFs is primarily controlled by intraovarian (autocrine/paracrine regulation) ligands (e.g., growth factors, cytokines, and gonadal steroids) even though it can be influenced by many extraovarian ligands (endocrine regulation) from different tissues including the endocrine glands (reviewed by Atwood and Meethala, 2016; Fig. 1-2). The control of folliculogenesis is, therefore, extremely complex because the aforementioned ligands act through distinct signaling pathways. These cell-signaling pathways do not act in isolation, but interact in various ways forming sophisticated information networks that respond to multiple, often opposing, stimuli. This connection may involve components that are common between pathways, as well as positive and negative feedback loops (Hunter, 2000). The main pathways currently studied are adenylate cyclase, MAPK / Erk, PI3K / Akt, phospholipase C, JAKS / STATS, SMADS and nuclear receptors. The ligands that regulate folliculogenesis act by binding to different types of receptors that activate one or more of these pathways leading to responses related to activation, survival, proliferation and follicular maturation (reviewed by Atwood and Meethala, 2016). The following sections describe the proposed mechanisms involved in the regulation of follicle growth from primordial to antral stage.

## Activation of primordial follicle

Follicular activation or recruitment is defined as the transition from primordial (quiescent follicle) to primary follicle (growing follicle). This process is marked by a rapid increase in oocyte volume that is accompanied by differentiation and proliferation of the surrounding pregranulosa cells into more cuboidal granulosa cells (reviewed by McLaughlin and McIver, 2009). The first visible sign that a primordial follicle is being activated is that some granulosa cells begin to change from a squamous to a cuboidal shape (reviewed by Figueiredo *et al*., 2011). The shape change is followed by the onset of DNA synthesis and mitosis in the granulosa cells. Activation is pituitary independent, and it probably is controlled by autocrine/paracrine mechanisms (reviewed by McLaughlin and McIver, 2009).

Culture of ovarian cortical tissue has been used to study primordial follicle activation *in vitro*. Kawamura *et al*. (2013) reported in human and mice that ovarian fragmentation increases actin polymerization and disrupts the Hippo signaling pathway, leading to an increase in expression of CCN growth factors. The name CCN is derived from major family members including cysteine-rich angiogenic protein (CYR61 or CCN1), connective tissue growth factor (CTGF or CCN2), and overexpression of nephroblastoma (NOV or CCN3). Secreted CCN2 and related factors promote primordial follicle growth *in vitro* (reviewed by Hsueh *et al*., 2015). Also, various growth factors (GDF9, BMP15, IGF1, kit ligand, BMP7, LIF, FGF2, FGF10 and TGFβ) and hormones (FSH, melatonin, growth hormone, oestradiol, and progesterone) are involved in the activation of primordial follicles *in vitro* (reviewed by Silva *et al*., 2016).

The mechanisms that regulate the initiation of growth of primordial follicle have been studied *in vivo* in murine species. It has been reported that growth factors, like KL, activate the phosphatidylinositol 3 kinase (PI3K) pathway in oocytes. Liu *et al*. (2007) showed that forkhead transcription factor 3 (FOXO3), serine/threonine kinase (Akt), phosphatase and tensin homolog deleted on chromosome 10 (PTEN) and glycogen synthase kinase 3A and 3B (GSK) are components involved in the PI3K pathway. FOXO3a is a downstream effector of the PTEN/PI3K/AKT pathway (Tran *et al*., 2003). In mouse ovaries, FOXO3a causes suppression of follicular activation, preserving the follicular reserve *pool* (Castrillon *et al*., 2003). A considerable proportion of the signaling mediated by PI3Ks converges at 3-phosphoinositide-dependent protein kinase-1 (PDK1). The PI3K-PDK1 cascade in mice oocytes regulates ovarian aging by regulating the survival of primordial follicles (Reddy *et al*., 2008). PTEN may act as a phosphoinositide-3 (PIP3)-phosphatase that antagonizes the activity of PI3K by dephosphorylating PIP3 to PIP2 (Maehama and Dixon, 1998). Oocyte-specific deletion of PTEN causes premature activation of mice primordial follicle pool, suggesting that the mammalian oocyte is the initiator of follicle activation and that the oocyte PTEN-PI3K pathway governs follicle activation through control of initiation of oocyte growth (Reddy *et al*., 2008). In bovine species, primordial follicle activation *in vitro* is associated with loss of the primordial follicle PTEN and cytoplasmic translocation of FOXO3 (Bromfield and Sheldon, 2013). Oocyte-specific loss of PTEN promotes in FOXO3 hyperphosphorylation, and FOXO3 nuclear export, which triggers the initiation of growth of primordial follicle, thus indicating that FOXO3 controls this step of follicular development (mice: John *et al*., 2008).

*In vivo* studies have shown that serine/threonine kinase mammalian target of rapamycin (mTORC) is another factor that controls primordial follicle activation since a suppression of its activity maintains the quiescence of mice primordial follicles (Reddy *et al*., 2008). These authors also reported that elevation of mTORC activity in the oocyte activates primordial follicles. In addition, the tumor suppressor tuberous sclerosis complex (TSC), which negatively regulates mTORC, functions in oocytes to maintain the quiescence of primordial follicles (Adhikari *et al*., 2010), Thus, the activity of both TSC and PTEN suppress mice follicular activation, but in distinct ways. They play an essential role in oocytes of mice primordial follicles to preserve the female reproductive lifespan (Adhikari *et al*., 2009).

*In vitro* studies have shown that various growth factors (GDF9, BMP15, IGF1, kit ligand, BMP7 LIF, FGF2, FGF10 and TGFβ) and hormones (FSH, melatonin, growth hormone, estradiol and progesterone) promote the activation of primordial follicles *in vitro* (reviewed by Silva *et al*., 2016). *In vitro* studies have shown that BMP15 increases follicle and oocyte diameters during culture of cortical tissues rich in primordial follicles (goat: Celestino *et al*., 2011). However, *in vivo* overexpression of *BMP15* gene in mice has no effect on the rate of primordial to primary follicle transition (McMahon *et al*., 2008). In addition, sheep containing homozygous mutations in this gene have normal primordial follicle activation, but folliculogenesis is arrested at the primary stage (Galloway *et al*., 2000). Recently, Zhao *et al*. (2018) showed that the growth factor activates phosphorylated mitogen-activated protein kinase3/1 (MAPK3/1) signaling in pregranulosa cells from mice to elevate mTORC1 signaling, leading to enhanced expression of Kit Ligand and subsequent activation of PI3K signaling in oocytes *in vitro*. Activation of this signaling pathway results in primordial follicle activation. Bisphenol, a chemical widely used in mineral water bottles and food- can linings, can initiate excessive premature activation of primordial follicles in mature mouse ovaries via the PTEN/PI3K/AKT signaling pathway by down regulating PTEN expression *in vivo* (Hu *et al*., 2017). Novella-Maestre *et al*. (2015) also showed that ovary-specific treatment with PTEN inhibitor enhances the activation mechanisms of primordial follicles, and also augments estradiol secretion in rat ovaries. In addition, Sun *et al*. (2005) treated mice and human ovaries with mTORC stimulators, i.e., phosphatidic acid (PA) and propranolol, and demonstrated that the stimulators increased activation of primordial follicles. On the other hand, gremlin-2 maintains the store of primordial follicles by suppressing Smad 1/5/8 signaling in the human ovary (Ikeda *et al*., 2016). However, the mechanisms of primordial follicle activation seem to be very complex, since it was reported that expression of 223 genes are down-regulated, while expression of 268 other genes are up-regulated in the oocytes during the human primordial-to-primary follicle transition (Ernst *et al*., 2017). The tyrosine kinase inhibitor imatinib mesylate blocks the activity of tyrosine kinase c-Kit, and is used as treatment for multiple cancers. It has also been proposed as an agent to prevent primordial follicle loss during chemotherapy based on its role as a c-Abl kinase inhibitor via PI3K/PTEN/Akt signaling pathways (Roness *et al*., 2014). However, it was shown recently that this compound down-regulates Kit ligand and GDF9 expression, with a delayed activation of rat primordial follicles *in vitro*. It is not clear if such a delay will affect the further follicular development (Asadi-Azarbaijani *et al*., 2017).

## Development of primary and secondary follicles

Once primary follicles are formed, the cuboidal granulosa cells begin to express FSH receptors probably through autocrine/paracrine mechanisms induced by granulosa-derived activin (reviewed by Silva *et al*., 2016). From the primary stage onwards, the oocyte begins to grow and differentiate as a result of a progressive increase in the level of oocyte RNA synthesis. Some oocyte genes including those encoding the zona pellucida (ZP) proteins (i.e. ZP1, ZP2 and ZP3) are transcribed and translated. During primary follicle development, gap junctions (intercellular channels composed of proteins called connexins-Cx that directly couple adjacent cells) are observed. Connexin 37 (C×37) is an oocyte-derived connexin that forms gap junctions between the oocyte and surrounding granulosa cells and has an obligatory role for folliculogenesis while C×43 is a major gap junction protein expressed in the granulosa cell layer (reviewed by Brito *et al*., 2014a). The communication between the granulosa cells and oocyte remains throughout folliculogenesis and is responsible for the synchronous expression of important activities. The acquisition of a theca layer (inner theca interna and outer theca externa) is an important event observed during the development of a secondary follicle. Some stromal cells in the inner layer express LH receptors (Young and McNeilly, 2010).

During the primary to secondary follicle transition, the granulosa cells proliferate to form multiple layers and thereby support oocyte growth (Richards and Pangas, 2010). During this early stage of folliculogenesis, oocytes begin to express abundant cell-cell communication proteins, including Cx37, N- and E- cadherin, and G-protein coupled receptors (Dharma *et al*., 2009). E- and N-cadherin are localized in the oocyte membrane and establish oocyte-granulosa cell contacts (Wang and Roy, 2010; Mora *et al*., 2012). In Cx37- deficient mice, folliculogenesis is arrested at the early antral stage, while in mice lacking Cx43, the follicles do not develop beyond the primary follicle stage, and oocyte growth is disrupted (Simon *et al*., 1997, Ackert *et al*., 2001). The mechanism to keep this bidirectional communication as well as to stimulate the primary to secondary follicles transition was recently reported by Jiang *et al*. (2017) in mice. These authors showed that geranylgeranyl diphosphate (GGPP), a metabolic intermediate involved in protein geranylgeranylation, is required to establish the oocyte-granulosa cell communication. In mice ovaries, the levels of GGPP and geranylgeranyl diphosphate synthase (GGPPS) in oocytes are increased during early folliculogenesis (Jiang *et al*., 2017). The depletion of GGPP in mouse oocytes impairs the proliferation of granulosa cells and disturbs the primary to secondary follicle transition. Regarding other mechanisms, GGPP depletion inhibits Rho GTPase geranylgeranylation and its GTPase activity, which is responsible for the accumulation of cell junction proteins in the oocyte cytoplasm. As a consequence, the physical connection between oocyte and granulosa cells is lost and the secretion of oocyte growth factors, like GDF9 is impaired (Jiang *et al*., 2017). It is important to consider that GDF9 knockout mice have follicular development arrested at the primary follicle stage (Dong *et al*., 1996). However, sheep with a homozygous mutation in GDF9 gene have normal follicular development up to the antral stages (McNatty *et al*., 2005). Additionally, GDF9 and BMP15 immunization experiments in sheep species caused a reduction in ovarian volume and abnormal follicular development beyond the primary follicle stage (Juengel *et al*., 2002).

**Figure 3:**
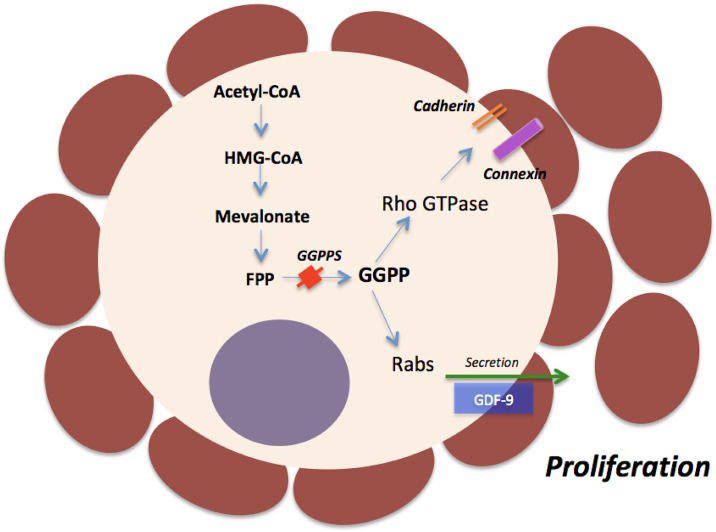
Mechanisms involving GGPP-mediated protein geranylgeranylation in the regulation of primary to secondary follicle transition.

Figure 3 shows the mechanisms by which GGPP-mediated protein geranylgeranylation regulates oocyte-granulosa cell communication and promotes the growth of primary follicles. Farnesyl diphosphate (FPP) is a metabolic intermediate of the mevalonate pathway that is catalyzed into geranylgeranyl diphosphate (GGPP) by geranylgeranyl diphosphate synthase (GGPPS). Then, GGPP activates Rho GTPase and Rab GTPase that stimulates the expression of cell junction proteins that are localized in the oocyte membrane to maintain the connection between granulosa cells and oocyte. This mechanism may be important for the secretion of growth factors that are synthesized exclusively by the oocyte, like GDF9 (mice: Jiang *et al*., 2017). These events stimulate proliferation of granulosa cells and primary to secondary follicle transition. Pires *et al*. (2013) also reported in mice and non-human primates that sperm acrosomal SLLP1 binding (ASTL) protein (ovastacin) starts expression during the primary to secondary transition, but not in primordial follicles, which suggest this zinc metalloprotease deserves consideration as a candidate to control primary to secondary follicles transition.

## Transition from preantral to antral follicle

The transition from secondary to tertiary follicle is marked by the appearance of a cavity in the granulosa cells named cavitation and/or beginning antrum formation. Early antrum formation is more likely to be controlled by autocrine/paracrine mechanisms rather than extra-ovarian factors like pituitary hormones such as FSH. As a matter of fact, cavitation occurs in hypophysectomized animals (Erickson, 1983) as well as in FSH-β-deficient mice (Kumar *et al*., 1998). In addition to gap junctions (Simon *et al*., 1997) two growth factors expressed in the follicle itself have been implicated in cavitation: activin and KIT ligand. Treating cultured granulosa cells with activin induced the formation of antrum-like cavity in rat follicles (Li *et al*., 1995). Blocking the action of the KIT ligand in the ovary prevents the formation of mouse antral follicles (Yoshida *et al*., 1997).

**Figure 4:**
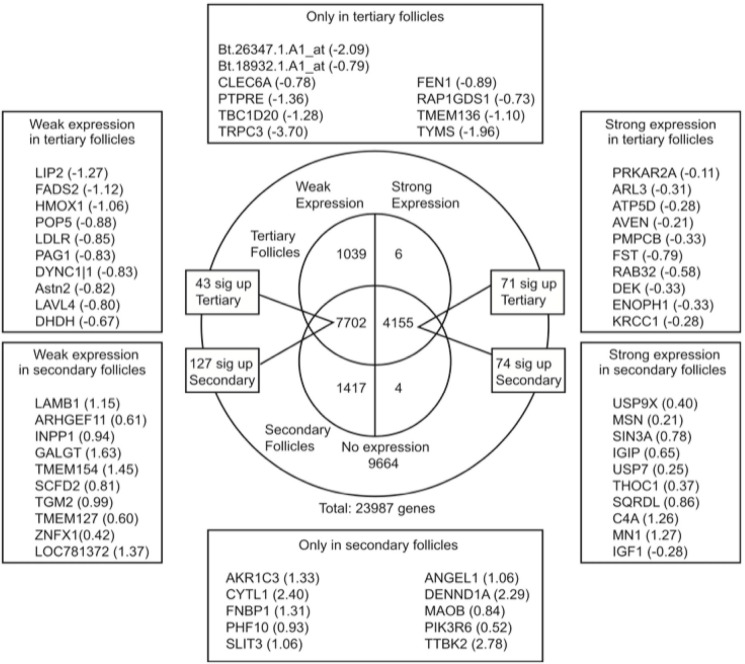
Overview of differential gene expression between secondary and tertiary ovarian follicles in a Venn diagram. Expressed genes were grouped into two categories weakly (left half) and strongly (right half) expressed. Strong expression means the expression level is above the genome average and weak expression means it is below the genome average. Top 10 representative genes from six categories were listed in boxes with their M value. M value was the log2 ratio of secondary follicle/tertiary follicle expression (Maglhães-Padilha et al., 2013).

Significant changes in gene expression pattern occur in the transition from late secondary to early tertiary follicles. To date, a study performed by our team investigated the temporal changes in transcriptional profiles of secondary and early antral (tertiary) follicles in caprine ovaries using microarray analysis. A total of 14,323 genes were hybridized with goat mRNAs while 9,664 genes were not. Of all the hybridized genes, 2,466 were stage-specific up- and down-regulated in the transition from secondary to early tertiary follicles (Fig. 4). Gene expression profiles showed that three major metabolic pathways (lipid metabolism, cell death, and hematological system) were significantly differentiated between the two follicle stages. (Magalhaes-Padilha *et al*., 2013).

Some studies have shown that the production of hyaluronan and proteoglycan by granulosa cells generates an osmotic gradient that enhances the formation of follicular fluid during transition from secondary to early tertiary follicles (Rodgers and Irving-Rodgers, 2010). These substances are osmotic solutes that act to increase the osmotic pressure inside the bovine follicle, resulting in fluid accumulation (Clarke *et al*., 2006). Versican and versican proteoglycans have been identified in the follicular fluid of various species, including bovine (McArthur *et al*., 2000; Clarke *et al*.; 2006) and human (Eriksen *et al*.; 1999), whereas perlecan protein have been identified in granulosa cells of bovine antral follicles (McArthur *et al*.; 2000; Princivalle *et al*.; 2001; Irving-Rodgers *et al*.; 2004). Familiari *et al*. (1987) reported that hyaluronan and proteoglycans, located on the apical side of granulosa cells, are then secreted into follicular fluid. Among the substances that promote the formation of antrum in bovine follicles, GDF9 increases the expression of versican and perlecan, as a consequence of a positive interaction with FSH. Both GDF9 and FSH also increase the *in vitro* expression of other antral fluid proteins, like versican and HAS2 (Vasconcelos *et al*., 2013).

## Contribution of *in vitro* follicle culture for the understanding of folliculogenesis

Immature oocytes are usually cultured *in vitro* enclosed in preantral (primordial, intermediate, primary and secondary) or antral (tertiary or early antral) follicles. This biotechnology is known as IVFC, and is mainly carried out in two forms: in situ, which means that ovarian follicles are cultured within the ovarian tissue, or in the isolated form (reviewed by Figueiredo *et al*., 2011). As mentioned before, the IVFC is an important tool to enhance the knowledge of the mechanisms involved in the control of ovarian folliculogenesis, including the development of PFs. The following sections discuss the major contribution of IVFC for the understanding of preantral follicle development focusing on domestic animals, especially ruminant species.

## Shared signaling pathways and similar effects of ligands on preantral follicle activation and growth in vitro

**Figure 5:**
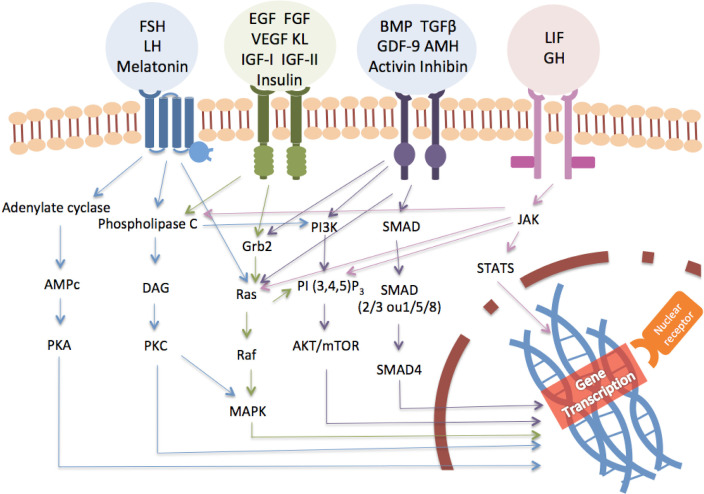
Signaling pathway of intra and extraovarian ligands in follicular development.

As in other tissues, when a ligand binds to its receptor in the ovary it will eventually affect gene expression, which in turn controls cell survival, proliferation and differentiation. As shown in Fig. 5 many intra and extraovarian ligands share the same signaling pathway. The addition of different ligands to a given control medium can result in similar effects. Studies have shown that the individual addition of many ligands to a determined culture medium improved in a concentration-depend manner the survival, activation and follicle and oocyte diameters after IVC of caprine ovarian tissue (reviewed by Figueiredo *et al*., 2011). However, the action of an individual ligand can be affected by the type of basic medium (TCM 199, MEM, etc.), the type of supplements present in this medium, as well as the volume of the medium and interval of its replenishment during IVFC (reviewed by Figueiredo *et al*., 2011). Consequently, care must be taken in the interpretation of the data and their possible translation to *in vivo* conditions.

## Gene expression in vivo and specific follicle requirements in vitro

Previously, temporal changes in transcriptional profiles of caprine secondary and early antral follicles have been reported. Such difference has a great impact on follicular requirements during IVC. Cadenas *et al*. (2017) investigated the effect of GH and VEGF added alone, sequentially, or in combination, in a medium supplemented with insulin at physiological concentration (10 ng/ml) on the IVC of large secondary preantral and early antral follicles from goats. Contrarily to PFs, in the IVC of early antral follicles, GH addition improved oocyte growth and maturation. It was previously shown that caprine secondary and early antral follicles present distinct gene expression (Magalhães-Padilha *et al*., 2013), which may result in different responses to some compounds. Based on RNA-seq technology, Bonnet *et al*. (2015) determined that the changes in gene expression that occur in both oocytes and granulosa cells respect a spatio-temporal process in sheep. In summary, depending on the follicular preantral phase the involved pathways are related to the acquisition of meiotic competence, migration and cellular organization, whereas in granulosa cells they are related to adhesion, formation of cytoplasmic projections and steroid synthesis. Also, changes in gene expression are more abundant in oocytes than in granulosa cells, with the transition between the primary to secondary follicles being the most active period. Knowledge of the important biomarkers of preantral follicular phase will allow the improvement and development of specific step-wise IVFC protocols.

## Heat stress and in vitro follicle development

Even though the impact of heat stress (HS) on antral follicles is well documented with *in vivo* studies, little is known about the influence of HS on the preantral phase of folliculogenesis. It was shown in bovine ovarian tissue *in vitro* that HS (41ºC) for 12 h induces early activation of primordial follicles with increased production of reactive oxygen species after 7 days IVFC (Paes *et al*., 2016). Furthermore, these authors showed that secondary PFs appear to be less sensitive to such stress, and that HS disrupts E2 and P4 secretion and reduces oocyte nuclear maturation of COCs from antral follicles grown *in vivo*.

## Follicle dominance (antral vs. preantral and influence of follicular fluid)

The importance of grouping PFs retrieved from domestic animals to increase growth rates in comparison with the culture of single follicles during IVFC obtained from pigs (Wu *et al*., 2001) and buffaloes (Gupta *et al*., 2002) was demonstrated. Duarte *et al*. (2010) observed that caprine secondary PFs cultured in groups (3 follicles/group) demonstrated enhanced viability, growth and antrum formation rates when compared to individually cultured follicles. However, co-culture of PFs with an early antral follicle had a detrimental effect on viability, antrum formation and production of oocytes for IVM. During *in vitro* culture of mouse follicles, Spears *et al*. (1996) demonstrated the occurrence of dominance in pairs of preantral follicles cultured in contact with each other until the antral stage of development. Probably, there is a specific interaction between adjacent follicles, which determines the success of dominant follicle development. Besides this, Duarte *et al*. (2012) showed that IVFC medium with follicular fluid from a dominant follicle enhanced caprine follicular survival, the maintenance of ultrastructure, as well as promoting follicular growth, meiosis resumption and early antrum formation. Follicular fluid is not only a source of hormones and growth factors. It was shown previously that follicular fluid increases the antioxidant capacity in *in vitro* cultured porcine granulosa cells, being also a source of anti-apoptotic factors (Santos *et al*., 2015). These authors also observed that such a protective effect is more robust when using follicular fluid from sows than from gilts.

## Extracellular matrix stiffness and primordial follicle activation

Usually, immature follicles gradually move from the rigid collagen-dense cortex zone to the less dense peri medullary region as they grow. To maintain the spherical structure of the follicle *in vitro*, 3D culture systems were developed in which the follicle floats in rotating tubes or inverted micro drops, or is encapsulated in a culture matrix, such as alginate. Although major advances in our understanding of follicle biology have been achieved with 2D attachment culture systems, recent studies have shown that 3D culture systems more closely mimic the physiological environment of the ovary, preserving follicular architecture and the interaction between somatic and germ cells (reviewed by Brito *et al*., 2014a). The use of IVC of isolated follicles allowed the evaluation of the importance of extracellular matrix rigidity (3D culture system) and of the contact between follicular cells in the activation of the primordial follicles and in the development of secondary follicles. Hornick *et al*. (2012) confirmed this fact using primate primordial follicles cultured in groups embedded in different alginate concentrations. They found that only the highest alginate tested concentration (2%) maintained the three-dimensional shape of the follicle and consequently an intimate contact between the follicular cells. This fact favored the activation and the *in vitro* growth that ends up in the production of secondary follicles. On the other hand, Brito *et al*. (2014b) increased oocyte maturation rates (59.52%) after culturing caprine secondary follicles in a less rigid alginate matrix (0.25%). These *in vitro* studies correlate with *in vivo* observation that primordial follicles are found primarily in the rigid and dense ovarian cortex rich in collagen, whereas the developing follicles are located closer to the medulla, in a less rigid environment.

## Role of oocyte and theca cells on in vitro follicle development

Interaction between the vascular theca and the avascular granulosa cells also play an important role in follicle development, not only because theca cells produce androgen that is aromatized in granulosa cells. Granulosa cells also regulate Theca cell steroidogenesis by providing steroids, growth factors, cytokines, and extracellular matrix, as shown in bovine (Parrott and Skinner, 2000). Liu *et al*. (2015) showed that co-culture of mouse theca cells with granulosa cells derived from PFs increased steroidogenesis in theca cells, together with the increase of mRNA expression of steroidogenic enzymes. Signaling between theca cells and oocyte also deserves attention when developing *in vitro* protocols. It is possible to observe migration of theca cells between co-cultured follicles (Campbell *et al*., 2013), which may contribute to the formation of mouse multiple oocyte follicles *in vitro* (Christensen *et al*., 2017) if these cells are not properly active.

## Antrum formation and the role of extra-ovarian factors

As the follicle diameter increases rapidly during late folliculogenesis, it was suggested that antrum is formed to overcome transport limitations (Gosden and Byattsmith, 1986). Both granulosa and thecal cells contribute to antrum formation by the synthesis and action of steroids and growth factors. However, extraovarian factors are necessary to allow PFs to form antrum *in vitro*, even though it is known that PFs are not gonadotropin dependent. Gutierrez *et al*. (2000) showed the importance of adding FSH to the culture medium to obtain an antrum in bovine cultured PFs. Araujo *et al*. (2014), however, showed also in bovine that the influence of extra-ovarian factors depends on the culture system. For instance, growth hormone addition in the culture medium was effective to stimulate estradiol synthesis only under 3D conditions.

## Final considerations

Follicular development from primordial to early antral stages is directly controlled by intra-ovarian ligands, and is influenced by many extra-ovarian ligands from different tissues including the endocrine glands. The control of early folliculogenesis is, therefore, extremely complex because several ligands act through distinct and interactive signalling pathways (for instance. adenylate cyclase, MAPK/Erk, PI3K/Akt, phospholipase C, JAKS/STATS, SMADS and nuclear receptors), forming sophisticated information networks that respond to multiple, often opposing, stimuli. The balance among different stimuli can lead to responses related to follicular survival or death as well as quiescence or activation (growth). It is important to emphasize that the distribution of the ligands and their corresponding receptors varies among follicular compartments (oocyte, granulosa and theca cells) and species (for instance between sheep and rodents) and significant changes in gene expression pattern among follicular categories have been reported. As a result, follicular requirements during early folliculogenesis seem to be stage-specific and species-specific. *In vivo* assessment of the mechanisms regulating follicle development is dependent on the animal species, and it can be affected by multiple factors including nutrition, breed, age, as well as individual variations. Therefore, especially for large domestic animals, interactions of factors and their effect on follicular development can be analyzed via IVFC studies. Hence, the IVFC technique enhances our understanding of the control of folliculogenesis. It also enhances the future use of a large number of immature oocytes enclosed in PFs in assisted reproductive technologies in humans as well as in others mammalian species. Last but not the least, it is important to highlight that the IVFC technique is a helpful tool to replace and reduce animal experimentation.

## References

[B1] Ackert CL, Gittens JE, O’Brien MJ, Eppig JJ, Kidder GM. (2001). Intercellular communication via connexin43 gap junctions is required for ovarian folliculogenesis in the mouse. Dev Biol.

[B2] Adhikari D, Flohr G, Gorre N, Shen Y, Yang H, Lundin E, Lan Z, Gambello MJ, Liu K. (2009). Disruption of Tsc2 in oocytes leads to overactivation of the entire pool of primordial follicles. Mol Hum Reprod.

[B3] Adhikari D, Zheng W, Shen Y, Gorre N, Hämäläinen T, Cooney AJ, Huhtaniemi I, Lan ZJ, Liu K. (2010). Tsc/mTORC1 signaling in oocytes governs the quiescence and activation of primordial follicles. Hum Mol Genet.

[B4] Araujo VR, Gastal MO, Wischral A, Figueiredo JR, Gastal EL. (2014). In vitro development of bovine secondary follicles in two- and three-dimensional culture systems using vascular endothelial growth factor, insulin-like growth factor-1, and growth hormone. Theriogenology.

[B5] Asadi-Azarbaijani B, Santos RR, Jahnukainen K, Braber S, van Duursen MBM, Toppari J, Saugstad OD, Nurmio M, Oskam IC (2017). Developmental effects of imatinib mesylate on follicle assembly and early activation of primordial follicle pool in postnatal rat ovary. Reprod Biol.

[B6] Atwood CS, Meethala SV. (2016). The spatiotemporal hormonal orchestration of human folliculogenesis, early embryogenesis and blastocyst implantation. Mol Cell Endocrinol.

[B7] Bonnet A, Servin B, Mulsant P, Mandon-Pepin B. (2015). Spatio-temporal gene expression profiling during in vivo early ovarian folliculogenesis: integrated transcriptomic study and molecular signature of early follicular growth. PLoS One.

[B8] Brito IR, Lima IM, Xu M, Shea LD, Woodruff TK, Figueiredo JR. (2014a). Three-dimensional systems for in vitro follicular culture: overview of alginate-based matrices. Reprod Fertil Dev.

[B9] Brito IR, Silva CM, Duarte AB, Lima IM, Rodrigues GQ, Rossetto R, Sales AD, Lobo CH, Bernuci MP, Rosa-E-Silva AC, Campello CC, Xu M, Figueiredo JR. (2014b). Alginate hydrogel matrix stiffness influences the in vitro development of caprine preantral follicles. Mol Reprod Dev.

[B10] Bromfield JJ, Sheldon IM. (2013). Lipopolysaccharide reduces the primordial follicle pool in the bovine ovarian cortex ex vivo and in the murine ovary in vivo. Biol Reprod.

[B11] Cadenas J, Leiva-Revilla J, Vieira LA, Apolloni LB, Aguiar FLN, Alves BG, Lobo CH, Rodrigues APR, Apgar GA, Smitz J, Figueiredo JR, Maside C. (2017). Caprine ovarian follicle requirements differ between preantral and early antral stages after IVC in medium supplemented with GH and VEGF alone or in combination. Theriogenology.

[B12] Campbell L, Trendell J, Spears N. (2013). Identification of cells migrating from the thecal layer of ovarian follicles. Cell Tissue Res.

[B13] Castrillon DH, Miao L, Kollipara R, Horner JW, DePinho RA. (2003). Suppression of ovarian follicle activation in mice by the transcription factor Foxo3a. Science.

[B14] Celestino JJH, Lima-Verde IB, Bruno JB, Matos MHT, Chaves RN, Saraiva MVA, Silva CMG, Faustino LR, Rossetto R, Lopes CAP, Donato MAM, Peixoto CA, Campello CC, Silva JRV, Figueiredo JR. (2011). Steady-state level of bone morphogenetic protein-15 in goat ovaries and its influence on in vitro development and survival of preantral follicles. Mol Cell Endocrinol.

[B15] Christensen AP, Peyrache E, Kaune H, Williams SA. (2017). Formation of multiple-oocyte follicles in culture. In vitro Cell Dev Biol Anim.

[B16] Clarke HG, Hope SA, Byers S, Rodgers RJ. (2006). Formation of ovarian follicular fluid may be due to the osmotic potential of large glycosaminoglycans and proteoglycans. Reproduction.

[B17] Dharma SJ, Modi DN, Nandedkar TD. (2009). Gene expression profiling during early folliculogenesis in the mouse ovary. Fertil Steril.

[B18] Dong J, Albertini DF, Nishimori K, Kumar TR, Lu N, Matzuk MM. (1996). Growth differentiation factor-9 is required during early ovarian folliculogenesis. Nature.

[B19] Duarte AB, Chaves RN, Araújo VR, Celestino JJ, Silva GM, Lopes CA, Tavares LM, Campelo CC, Figueiredo JR. (2010). Follicular interactions affect the in vitro development of isolated goat preantral follicles. Zygote.

[B20] Duarte AB, Araújo VR, Chaves RN, Silva GM, Magalhães-Padilha DM, Satrapa RA, Donato MA, Peixoto CA, Campello CC, Matos MH, Barros CM, Figueiredo JR. (2012). Bovine dominant follicular fluid promotes the in vitro development of goat preantral follicles. Reprod Fertil Dev.

[B21] Erickson GF. (1983). Primary cultures of ovarian cells in serum-free medium as models of hormone-dependent differentiation. Mol Cell Endocrinol.

[B22] Eriksen GV, Carlstedt I, Morgelin M, Uldbjerg N, Malmstrom A. (1999). Isolation and characterization of proteoglycans from human follicular fluid. Biochem J.

[B23] Ernst EH, Grøndahl ML, Grund S, Hardy K, Heuck A, Sunde L, Franks S, Andersen CY, Villesen P, Lykke-Hartmann K. (2017). Dormancy and activation of human oocytes from primordial and primary follicles: molecular clues to oocyte regulation. Hum Reprod.

[B24] Familiari G, Nottola SA, Motta PM. (1987). Focal cell contacts detected by rutheniumred, TritonX100 and saponin in the granulosa cells of mouse ovary. Tissue Cell.

[B25] Figueiredo JR, Celestino JJH, Faustino LR, Rodrigues APR. (2011). In vitro culture of caprine preantral follicles: advances, limitations and prospects. Small Rumin Res.

[B26] Galloway SM, McNatty KP, Cambridge LM, Laitinen MPE, Juengel JL, Jokiranta TS, McLaren RJ, Luiro K, Dodds KG, Montgomery GW, Beattie AE, Davis GH, Ritvos O. (2000). Mutations in an oocyte-derived growth factor gene (bmp15) cause increased ovulation rate and infertility in a dosage-sensitive manner. Nat Genet.

[B27] Gosden RG, Byattsmith JG. (1986). Oxygen concentration gradient across the ovarian follicular epithelium - model, predictions and implications. Hum Reprod.

[B28] Gupta PSP, Nandi S, Ravindranatha BM, Sarma PV. (2002). In vitro culture of buffalo (Bubalus bubalis) preantral follicles. Theriogenology.

[B29] Gutierrez CG, Ralph JH, Telfer EE, Wilmut I, Webb R. (2000). Growth and antrum formation of bovine preantral follicles in long-term culture in vitro. Biol Reprod.

[B30] Hornick JE, Duncan FE, Shea LD, Woodruff TK. (2012). Isolated primate primordial follicles require a rigid physical environment to survive and grow in vitro. Hum Reprod.

[B31] Hsueh AJ, Kawamura K, Cheng Y, Fauser BC. (2015). Intraovarian control of early folliculogenesis. Endocr Rev.

[B32] Hu Y, Yuan DZ, Wu Y, Yu LL, Xu LZ, Yue LM, Liu L, Xu WM, Qiao XY, Zeng RJ, Yang ZL, Yin WY, Ma YX, Nie Y. (2017). Bisphenol A initiates excessive premature activation of primordial follicles in mouse ovaries via the PTEN signaling pathway. Reprod Sci.

[B33] Hunter T. (2000). Cell, signaling-2000 and beyond. Cell Press.

[B34] Ikeda Y, Hasegawa A, Tsubamoto H, Wakimoto Y, Kumamoto K, Shibahara H. (2016). Effects of gremlin-2 on the transition of primordial follicles during early folliculogenesis in the human ovary. Eur J Obstet Gynecol Reprod Biol.

[B35] Irving-Rodgers HF, Harland ML, Rodgers RJ. (2004). A novel basal lamina matrix of the stratified epithelium of the ovarian follicle. Matrix Biol.

[B36] Jiang C, Diao F, Sang YJ, Xu N, Zhu RL, Wang XX, Chen Z, Tao WW, Yao B, Sun HX, Huang XX, Xue B, Li CJ. (2017). GGPP-mediated protein geranylgeranylation in oocyte is essential for the establishment of oocyte-granulosa cell communication and primary-secondary follicle transition in mouse ovary. PLoS Genet.

[B37] Jimenez CR, Araújo VR, Penitente-Filho JM, Azevedo JL, Silveira RG, Torres CAA (2016). The base medium affects ultrastructure and survival of bovine preantral follicles cultured in vitro. Theriogenology.

[B38] John GB, Gallardo TD, Shirley LJ, Castrillon DH. (2008). Foxo3 is a PI3K dependent molecular switch controlling the initiation of oocyte growth. Dev Biol.

[B39] Juengel JL, Hudson NL, Heath DA, Smith P, Reader KL, Lawrence SB, O’Connel Laitinen MP, Cranfield M, Groome NP, Ritvos O, McNatty KP (2002). Growth differentiation factor 9 and bone morphogenetic protein 15 are essential for ovarian follicular development in sheep. Biol Reprod.

[B40] Kawamura A, Cheng Y, Suzuki N, Deguchi M, Sato Y, Takae S, Chi-hong Ho, Kawamura N, Tamura M, Hashimoto S, Sugishita Y, Morimoto Y, Hosoi Y, Yoshioka N, Ishizuka B, Hsueh AJ (2013). Hippo signaling disruption and Akt stimulation of ovarian follicles for infertility treatment. Proc Natl Acad Sci USA.

[B41] Kumar TR, Low MJ, Matzuk MM. (1998). Genetic rescue of follicle-stimulating hormone beta-deficient mice. Endocrinology.

[B42] Li R, Phillips DM, Mather JP. (1995). Activin promotes ovarian follicle development in vitro. Endocrinology.

[B43] Liu L, Rajareddy S, Reddy P, Jagarlamudi K, Du C, Shen Y, Guo Y, Boman K, Lundin E, Ottander U, Selstam G, Liu K. (2007). Phosphorylation and inactivation of glycogen synthase kinase-3 by soluble kit ligand in mouse oocytes during early follicular development. J Mol Endocrinol.

[B44] Liu X, Qiao P, Jiang A, Jiang J, Han H, Wang L, Ren C. (2015). Paracrine regulation of steroidogenesis in theca cells by granulosa cells derived from mouse preantral follicles. Biomed Res Int.

[B45] Maehama T, Dixon JE. (1998). The tumor suppressor, PTEN/ MMAC1, dephosphorylates the lipid second messenger, phospha-tidylinositol 3, 4, 5-trisphosphate. J Biol Chem.

[B46] Magalhães-Padilha DM, Geisler-Lee J, Wischral A, Gastal MO, Fonseca GR, Eloy YR, Geisler M, Figueiredo JR, Gastal EL. (2013). Gene expression during early folliculogenesis in goats using microarray analysis. Biol Reprod.

[B47] McArthur ME, Irving-Rodgers HF, Byers S, Rodgers RJ. (2000). Identification and immunolocalization of decorin, versican, perlecan, nidogen, and chondroitin sulfate proteoglycans in bovine small-antral ovarian follicles. Biol Reprod.

[B48] McLaughlin EA, McIver SC (2009). Awakening the oocyte: controlling primordial follicle development. Reproduction.

[B49] McMahon HE, Hashimoto O, Mellon PL, Shimasaki S. (2008). Oocyte-specific overexpression of mouse bone morphogenetic protein-15 leads to accelerated folliculogenesis and an early onset of acyclicity in transgenic mice. Endocrinology.

[B50] McNatty KP, Galloway SM, Wilson T, Smith P, Hudson NL, O'Connell A, Bibby AH, Heath DA, Davis GH, Hanrahan JP, Juengel JL. (2005). Physiological effects of major genes affecting ovulation rate in sheep. Genet Sel Evol.

[B51] Mora JM, Fenwick MA, Castle L, Baithun M, Ryder TA, Mobberley M, Carzaniga R, Franks S, Hardy K. (2012). Characterization and significance of adhesion and junction-related proteins in mouse ovarian follicles. Biol Reprod.

[B52] Novella-Maestre E, Herraiz S, Rodríguez-Iglesias B, Díaz-García C, Pellicer A. (2015). Short-term PTEN inhibition improves in vitro activation of primordial follicles, preserves follicular viability, and restores AMH levels in cryopreserved ovarian tissue from cancer patients. PLoS One.

[B53] Paes VM, Vieira LA, Correia HHV, Sa NAR, Moura AAA, Sales AD, Rodrigues APR, Magalhães-Padilha DM, Santos FW, Apgar GA, Campello CC, Camargo LSA, Figueiredo JR. (2016). Effect of heat stress on the survival and development of in vitro cultured bovine preantral follicles and on in vitro maturation of cumulus-oocyte complex. Theriogenology.

[B54] Parrott JA, Skinner MK. (2000). Kit ligand actions on ovarian stromal cells: effects on theca cell recruitment and steroid production. Mol Reprod Dev.

[B55] Pires ES, Hlavin C, Macnamara E, Ishola-Gbenla K, Doerwaldt C, Chamberlain C, Klotz K, Herr AK, Khole A, Chertihin O, Curnow E, Feldman SH, Mandal A, Shetty J, Flickinger C, Herr JC. (2013). SAS1B protein [ovastacin] shows temporal and spatial restriction to oocytes in several eutherian orders and initiates translation at the primary to secondary follicle transition. Dev Dyn.

[B56] Princivalle M, Hasan S, Hosseini G, Agostini AI. (2001). Anticoagulant heparan sulfate proteoglycans expression in the rat ovary peaks in preovulatory granulosa cells. Glycobiology.

[B57] Reddy P, Liu L, Adhikari D, Jagarlamudi K, Rajareddy S, Shen Y, Du C, Tang W, Hämäläinen T, Peng SL, Lan ZJ, Cooney AJ, Huhtaniemi I, Liu K. (2008). Oocyte-specific deletion of PTEN causes premature activation of the primordial follicle pool. Science.

[B58] Richards JS, Pangas SA. (2010). The ovary: basic biology and clinical implications. J Clin Invest.

[B59] Rodgers RJ, Irving-Rodgers HF. (2010). Formation of the ovarian follicular antrum and follicular fluid. Biol Reprod.

[B60] Roness H, Kalich-Philosoph L, Meirow D. (2014). Prevention of chemotherapy-induced ovarian damage: possible roles for hormonal and non-hormonal attenuating agents. Hum Reprod Update.

[B61] Santos RR, Schoevers EJ, Wu X, Roelen BAJ, Fink-Gremmels J. (2015). The protective effect of follicular fluid against the emerging mycotoxins alternariol and beauvericin. World Mycotoxin J.

[B62] Silva JRV, van den Hurk R, Matos MHT, Santos RR, Pessoa C, Moraes MO, Figueiredo JR. (2004). Influences of FSH and EGF on primordial follicles during in vitro culture of caprine ovarian cortical tissue. Theriogenology.

[B63] Silva JRV, van den Hurk R, Figueiredo JR. (2016). Ovarian follicle development in vitro and oocyte competence: advances and challenges for farm animals. Domest Anim Endocrinol.

[B64] Simon AM, Goodenough DA, Li E, Paul DL. (1997). Female infertility in mice lacking connexin 37. Nature.

[B65] Spears N, De Bruin JP, Gosden RG. (1996). The establishment of follicular dominance in co-cultured mouse ovarian follicles. J Reprod Fertil.

[B66] Sun X, Su Y, He Y, Zhang J, Liu W, Zhang H, Hou Z, Liu J, Li J. (2005). New strategy for in vitro activation of primordial follicles with mTOR and PI3K stimulators. Cell Cycle.

[B67] Tran H, Brunet A, Griffith EC, Greenberg ME. (2003). The many forks in FOXO's Road. Sci STKE.

[B68] Vasconcelos GL, Saraiva MV, Costa JJ, Passos MJ, Silva AW, Rossi RO, Portela AM, Duarte AB, Magalhães-Padilha DM, Campelo CC, Figueiredo JR, van den Hurk R, Silva JR. (2013). Effects of growth differentiation factor-9 and FSH on in vitro development, viability and mRNA expression in bovine preantral follicles. Reprod Fertil Dev.

[B69] Wang C, Roy SK. (2010). Expression of E-cadherin and N-cadherin in perinatal hamster ovary: possible involvement in primordial follicle formation and regulation by follicle-stimulating hormone. Endocrinology.

[B70] Wu J, Emery BR, Carrell DT. (2001). In vitro growth, maturation, fertilization, and embryonic development of oocytes from porcine preantral follicles. Biol Reprod.

[B71] Yoshida H, Takakura N, Kataoka H, Kunisada T, Okamura H, Nishikawa SI. (1997). Stepwise requirement of c-kit tyrosine kinase in mouse ovarian follicle development. Dev Biol.

[B72] Young JM, McNeilly AS. (2010). Theca: the forgotten cell of the ovarian follicle. Reproduction.

[B73] Zhao Y, Zhang Y, Li J, Zheng N, Xu X, Yang J, Xia G, Zhang M. (2018). MAPK3/1 participates in the activation of primordial follicles through mTORC1-KITL signaling. J Cell Physiol.

